# Trade-Off between COVID-19 Pandemic Prevention and Control and Economic Stimulus

**DOI:** 10.3390/ijerph192113956

**Published:** 2022-10-27

**Authors:** Fangfang Liu, Zheng Ma, Ziqing Wang, Shaobo Xie

**Affiliations:** 1 School of Marxism, Chang’an University, Xi’an 710064, China; 2 School of Automobile, Chang’an University, Xi’an 710064, China; 3 NIT-O2S, UTBM, University Bourgogne Franche-Comté, 91110 Belfort, France

**Keywords:** COVID-19, pandemic prevention and control, economic stimulus, trade-off

## Abstract

The coronavirus disease 2019 (COVID-19) pandemic has posed a severe threat to public health and economic activity. Governments all around the world have taken positive measures to, on the one hand, contain the epidemic spread and, on the other hand, stimulate the economy. Without question, tightened anti-epidemic policy measures restrain people’s mobility and deteriorate the levels of social and economic activity. Meanwhile, loose policy measures bring little harm to the economy temporarily but could accelerate the transmission of the virus and ultimately wreck social and economic development. Therefore, these two kinds of governmental decision-making behaviors usually conflict with each other. With the purpose of realizing optimal socio-economic benefit over the full duration of the epidemic and to provide a helpful suggestion for the government, a trade-off is explored in this paper between the prevention and control of the epidemic, and economic stimulus. First, the susceptible–infectious–recovered (SIR) model is introduced to simulate the epidemic dynamics. Second, a state equation is constructed to describe the system state variable—the level of socio-economic activity dominated by two control variables. Specifically, these two variables are the strengths of the measures taken for pandemic prevention and control, and economic stimulus. Then, the objective function used to maximize the total socio-economic benefit over the epidemic’s duration is defined, and an optimal control problem is developed. The statistical data of the COVID-19 epidemic in Wuhan are used to validate the SIR model, and a COVID-19 epidemic scenario is used to evaluate the proposed method. The solution is discussed in both static and dynamic strategies, according to the knowledge of the epidemic’s duration. In the static strategy, two scenarios with different strengths (in terms of anti-epidemic and economic stimulus measures) are analyzed and compared. In the dynamic strategy, two global optimization algorithms, including the dynamic programming (DP) and Pontryagin’s minimum principle (PMP), respectively, are used to acquire the solutions. Moreover, a sensitivity analysis of model parameters is conducted. The results demonstrate that the static strategy, which is independent of the epidemic’s duration and can be easily solved, is capable of finding the optimal strengths of both policy measures. Meanwhile, the dynamic strategy, which generates global optimal trajectories of the control variables, can provide the path that leads to attaining the optimal total socio-economic benefit. The results reveal that the optimal total socio-economic benefit of the dynamic strategy is slightly higher than that of the static strategy.

## 1. Introduction

The COVID-19 pandemic has continued for three years worldwide, and has been recognized as one of the most severe public health crises to emerge in the history of the human race. The pandemic poses a huge threat to public health. According to statistics from the World Health Organization (WHO), as of October 2022, the confirmed cases of and deaths due to COVID-19 exceed 615 million and 6.5 million, respectively [1]. At the same time, the pandemic has negatively impacted many countries’ social and economic development. The International Monetary Fund (IMF) reports that in 2020, when the epidemic raged out of control, the global economy contracted by about 3.1% [2]. There is broad agreement that such a pandemic has led to the decay of the global economy. Unfortunately, this COVID-19 epidemic will apparently not be over in the next few years [3], because of the acute, occult and highly contagious character of the virus.

Policy makers all over the world have taken positive measures to cope with and restrict the spread of this epidemic [4,5]. Examples include the mandated wearing of masks in public places, maintaining social distancing, home isolation, business closures, and even full lockdowns in some local areas [6]. In China, the government adopted even stricter policies, i.e., the zero-case zone strategy, to rapidly stop the virus from spreading in local areas [7]. Meanwhile, the vaccination policy has been pushed forward in many countries [8]. These prevention and control strategies, especially at the beginning of the outbreak, have restrained people’s mobility and hampered economic activity, resulting in huge socio-economic losses. When implementing anti-epidemic policies, many authorities hope to help their economy to recover, and have therefore implemented various stimulus policy measures. Included were fiscal, financial and monetary strategies, particularly quantitative easing, which were implemented to activate the market and avoid an economic recession [9,10].

However, the strength of epidemic prevention and control measures and economic stimulus measures are usually two conflicting decision behaviors [11]. Stringent anti-epidemic policy measures, such as the static management of local zones in some Chinese cities, confine both production and consumption, impact logistics and transportation and unavoidably damage the area’s socio-economic vitality [12]. On the other hand, a looser anti-epidemic policy would be more likely to result in fast and widespread virus transmission, eventually damaging the socio-economic benefit such policies are intended to have. In other words, the strengths of the pandemic prevention and control measures on the one hand the economic stimulus measures on the other hand influence each other. The differences in the strengths of the anti-epidemic and economic boost measures are like the two sides of a seesaw, where a preference for any part of either side would destroy the balance, and ultimately weaken the total benefits of the two players on that seesaw. Seeking the optimal total socio-economic benefit over the entire duration of the epidemic, a trade-off between the two kinds of governmental decision behaviors is explored in this paper.

## 2. Literature Review

To curb the spread of the COVID-19 virus, governments all around the world have taken a variety of intervention measures. These measures include social distancing, school closures, quarantine, lockdown or stay at home measures and restrictions on business, mass gatherings, travel and external borders, as well as securing health resources and vaccine and drug research and development [13,14]. At the same time, many economic intervention policies have been launched to mitigate the economic impact and recover economic activity.

### 2.1. Economic Impact and Intervention Policies

Apart from the serious damage to public health, the outbreak of the pandemic has had an extensive negative impact on economic activity. The affected fields include the labor market, the supply chain and the consumption market, and the sectors cover the transport, logistic, tourism and service industries. These measures have led to mass layoffs and unemployment, and huge economic losses [15]. The mechanism for macroeconomic impact includes three channels: (1) the direct impact, which is relevant to goods and services, (2) the indirect impact related to financial market shocks and the real economy and (3) supply chain disruption [16,17]. Several scholars have assessed the economic losses caused by the COVID-19 pandemic. The quantitative calculation shows that the COVID-19 pandemic has led to a 12.75% reduction in industrial production, and a 17% drop in service employment [18,19]. Cutler et al. [20] estimated that the economic cost of the COVID-19 crisis in the U.S. in 2020 was more than 16 trillion dollars, or nearly 90% of the country’s annual gross domestic product. Moreover, special economic models or approaches have suggested how to quantify the economic impact of the COVID-19 pandemic. For example, Tang et al. [21] employed the computable general equilibrium model to evaluate the influence of COVID-19 on China’s economy from the perspectives of the direct impact, international trade and work resources. Fadinger et al. [22] used an input–output model to investigate the effect of the pandemic across a regional economy. Hector et al. [23] simulated the impact of the COVID-19 pandemic by using a post-Keynesian approach.

To eliminate or at least reduce the damage caused by the pandemic, many authorities have adopted targeted policy measures to stimulate the economy. For example, Makin et al. [24] found that global central governments and banks tend to implement extensive fiscal and current measures to increase fiscal output, remove taxes and transfer payments to help overcome economic hardships. Pavle et al. [25] analyzed the EU’s fiscal policies, and found that increasing public investment can be an effective policy in tackling economic decay. The Chinese government adopted a series of policies, including fiscal, monetary and tax measures, as well as the strategy of providing additional investment for infrastructure construction [26]. Some local authorities even directly provided special anti-epidemic funds, shopping vouchers and unemployment compensation taxes for citizens [27].

### 2.2. Relationship between Epidemic Containment and Economic Development

The government intervention measures, from the perspectives of epidemic prevention and control and economic development, have been extensively discussed, and their relationship has also been explored. These studies can be categorized into qualitative and quantitative types. The qualitative ones mainly elaborate the interactive relationship between two governmental decision-making behaviors from the actual effect and statistical data. For example, Gong et al. [28] reviewed the instances of typical epidemics, such as the SARS, H1N1 and Ebola epidemics, as well as their economic influences. The authors argued that to realize a balancing act in the fight against COVID-19 in China, accurate and transparent disclosure of information plays a critical role. In addition, the importance of the information needed for epidemic control lies in three aspects, namely the collection, processing and dissemination of epidemic information. Lin [29] explained the relationship between epidemic prevention and control and economic development in China, and demonstrated that the epidemic prevention and control measures provide a strong guarantee with regard to promoting economic development, especially with regard to the growth of foreign trade. In various quantitative studies, the government intervention policies used in economic and epidemic control have been modeled by using epidemic dynamics and economic theory. An equilibrium problem or optimization problem between two governmental behaviors can also be formulated. For example, Eichenbaum et al. [30] examined the connection between economic decisions—particularly cutbacks of consumption and work—and halting the spread of the virus. The study concluded that removing anti-epidemic measures too early will result in large benefits in the short term, but will not achieve consistent benefits in the long run. Yin et al. [31] studied the link between, on the one hand, government intervention and the spread of the pandemic and, on the other hand, economic development under conditions of public health emergency shocks. The results also demonstrate that strict anti-virus policy measures have a limited effect in recovering from the harmful impact of the public health emergencies caused by the highly contagious virus. Xiang et al. [32] found that although China’s public health policy at the initial stage was conductive to both virus containment and economic recovery, its marginal efficiency gradually waned. Only if the pandemic is controlled effectively will the economic stimulation policy function properly.

Although epidemic containment and economic intervention measures have been addressed, their quantitative relationship needs to be further discussed from the perspective of a whole governmental decision-making system. Moreover, most governmental policy measures have been designed according to the current epidemic condition and economic activity. To be precise, they are based on the short-term epidemic situation and not intentionally planned to operate over the whole process of an epidemic wave. For this reason, the total optimum socio-economic benefit cannot be maximized for the duration of the pandemic. In fact, a COVID-19 epidemic wave may last for many days. Therefore, these short-term or temporary policies may incur the imbalance of both decision behaviors. Epidemic prevention and control that is too rigorous can rapidly contain the virus’s transmission but may induce sharp decays in local economies. For example, the National Health Commission in China recently called on several local governments to set reasonable anti-epidemic measures and to remove unnecessary or even excessive anti-epidemic policy measures. Conversely, strategies to prevent and control the spread of the virus that are too relaxed, together with tightened economic stimulus packages, could lead to a recurrence of the epidemic. Clearly, this would not achieve the optimal governmental total socio-economic benefit over the whole duration of the pandemic. Therefore, realizing a balance between both types of governmental decision-making behaviors throughout the duration of the epidemic is critically important.

Moreover, governmental decision-making behaviors are usually affected by many factors, including the dominant regime, culture and industrialization level, all of which vary from one country to another. The common mechanism-based model typically employed to quantify the socio-economic activity and benefit cannot cover all of the real-world factors, and this leads to a gap between the model and reality. Therefore, this paper tries to describe the level of socio-economic activity from the perspectives of the positive and negative effects generated by governmental decision-making behaviors. This approach can avoid the complexity and drawbacks of the mechanism-based model.

In addition, optimal control theory, which is capable of describing a system’s evolution and dynamically optimizing two interactive decision-making behaviors from the global horizon, is suitable for studying the trade-off between epidemic prevention and control and economic stimulus measures. Despite the fact that several mathematical methods, such as the multiple-objective optimization method, have been suggested as a way to deal with the relationship between two intervention behaviors, optimal control theory, to the best of our knowledge, has not yet been employed. To fill this research gap, the optimal control theory is presented as a method to solve the dynamic balance of two different kinds of governmental behaviors.

The contributions of this work are from the following aspects. (i) This paper quantitatively investigates the trade-off between public health and economic stimulus measures, based on the optimal control theory. Specifically, global optimization algorithms, including the dynamic programming algorithm and Pontryagin’s minimum principle algorithm, are applied to solve the optimal control problem. This interdisciplinary research work refers to many fields, such as public health, epidemic dynamics, economics and control theory. (ii) The epidemic dynamics simulated by the SIR model are incorporated into the optimal control problem; the four-order Runge–Kutta method is also used to solve this model to realize the forecast of the pandemic’s infections. Moreover, the statistical data of Wuhan during the COVID-19 epidemic wave are used to validate the model. (iii) Traditional mechanism-based models used to describe governmental decision-making behaviors usually involve many real-world factors. This is not convenient for quantification, so the mathematical model in this study is developed based on the positive and negative effects of governmental behaviors. (iv) The solution of the optimal control problem is discussed through two strategies, namely static and dynamic strategies, based on each strategy’s unique advantages. The former can easily acquire the solution, despite the expense of losing a minimal amount of accuracy; the latter is capable of generating the global optimal path of decision-making behaviors.

The remainder of this paper is organized as follows. First, the epidemic dynamic model is introduced. Second, the system dynamic model is established in order to describe the relationship between governmental decision-making behaviors and the level of socio-economic activity. Then, the objective function is defined. The optimal control problem is developed and solved using both the static and dynamic strategies. Specially, for the dynamic strategies, the dynamic programming and Pontryagin’s minimum principle are utilized and compared. After that, the epidemic dynamic model is validated, the results of both strategies are discussed and the parameter sensitivity is analyzed. Finally, the study’s conclusions are drawn, and the study’s limitations are pointed out.

## 3. Epidemic Dynamic Model

An epidemic dynamic model can provide a critical reference for designing appropriate intervention policies. Many mathematical methods have been used to simulate the virus’s transmission mechanism. The widely used model is the SIR model [33,34], which can simulate the essential process of the disease’s spread over time. For different viral types, the susceptible–infectious–susceptible (SIS) model and SIR model’s variant models have also been applied [35]. Meanwhile, the agent-based computational models [36] and Bayesian approach [37] present promising capability in terms of modeling to contain pandemics. With enormous available data about the disease, the data-driven models have also been employed by using the artificial neural network [38]. As the SIR model is capable of reflecting the underlying mechanism of the virus spread over time, and does not involve too many parameters to be calibrated, the SIR model is selected here to predict the infection population of the COVID-19 pandemic.

For the SIR model, the population is divided into three fractions, namely the susceptible, infected and recovered individuals. The differential equation of this model is as follows [34]:

(1) $$\{\begin{array}{l} \dot{S}\left(t\right)=-\beta S\left(t\right)I\left(t\right)\\ \dot{I}\left(t\right)=\beta S\left(t\right)I\left(t\right)-\gamma I\left(t\right)\\ \dot{R}\left(t\right)=\gamma I\left(t\right) \end{array}$$

where *t* is the time variable; *S*, *I* and *R* denote the susceptible, infected and recovered individuals, respectively; parameter β is the infective rate, which denotes the average number of individuals that one infected individual will infect per time unit; and γ denotes the recovery rate.

The four-order Runge–Kutta algorithm is employed to obtain the numerical solution to the differential equations generated by the SIR model [39]:

(2) $$\{\begin{array}{c} S\left(t+1\right)=S\left(t\right)+\frac{h}{6}\left(a_{11}+a_{21}+a_{31}+a_{41}\right)\\ I\left(t+1\right)=I\left(t\right)+\frac{h}{6}\left(a_{12}+a_{22}+a_{32}+a_{42}\right)\\ R\left(t+1\right)=R\left(t\right)+\frac{h}{6}\left(a_{13}+a_{32}+a_{33}+a_{43}\right) \end{array}$$

where h is fixed-step and the coefficients have the form of:

(3) $$\{\begin{array}{l} a_{1i}=f_{i}\left(S,I\right)\\ a_{2i}=f_{i}\left(S\left(t\right)+\frac{h}{2}a_{11},I\left(t\right)+\frac{h}{2}a_{12}\right)\\ a_{3i}=f_{i}\left(S\left(t\right)+\frac{h}{2}a_{21},I\left(t\right)+\frac{h}{2}a_{22}\right)\\ a_{4i}=f_{i}\left(S\left(t\right)+\frac{h}{2}a_{31},I\left(t\right)+\frac{h}{2}a_{32}\right) \end{array}$$

where the index *i* = 1, 2, 3.

## 4. Optimal Control Problem

### 4.1. System Dynamic Model

Governmental decision-making behaviors and their corresponding costs and benefits can be regarded as a dynamic system, where the state variable is dominated by the control variable. The general dynamic equation is formulated as:

(4) $$\dot{x}\left(t\right)=f\left(x\left(t\right),u\left(t\right)\right)$$

where x is the state variable, $\dot{x}$ is the derivative of x, t is the time variable, u is the control variable and f is the system dynamic equation.

When dealing with the emergence of COVID-19, government decision-making behaviors can be mainly divided into two types. One behavior is for pandemic prevention and control, and the other behavior is for economic stimulus. On the one hand, rational policy makers take positive measures to prevent the pandemic. On the other hand, they will also make efforts to help with economic recovery to ease the epidemic shock effects. In this study, the level of socio-economic activity is selected as the state variable, and the strengths of the pandemic prevention and control measures and economic stimulus measures are considered as two control variables. Moreover, the number of infections, which is considered as the critical indicator used to evaluate the epidemic situation, directly influences socio-economic activity. In short, the investment in anti-epidemic measures and the increasing number of infections have a negative effect on socio-economic activity, while the economic stimulus measures have a positive effect.

From the perspective of the effectiveness of decision-making behaviors, the system state equation can be described as:

(5) $$\dot{x}\left(t\right)=\alpha_{s}E_{s}\left(t\right)-\alpha_{p}E_{p}\left(t\right)-k_{i}N_{i}\left(t\right)-\delta x\left(t\right)$$

where the state variable x is the level of socio-economic activity; the two control variables $E_{p}$ and $E_{s}$ denote the strength of epidemic prevention and control and economic stimulus, respectively; $N_{i}$ denotes the predicted number of infections; $\alpha_{p}$ and $\alpha_{s}$ are the effective coefficients of the two control variables, respectively; $k_{i}$ is the effective coefficient from the number of infected people; and δ is the decaying factor of the state variable.

### 4.2. Optimal Control Problem

The government investments in the anti-epidemic and economic stimulus measures aim to provide socio-economic benefits, including benefits from economic recovery and development, human health protection and the improvement of the government’s public image. Correspondingly, government investments come at a cost. In this study, the governmental cost is assumed to be a quadratic function with respect to the government’s investments [40]. Then, the objective function can be defined as:

(6) $$J=\underset{E_{p},E_{s}}{\underset{︸}{max}}\int_{0}^{T}\left(\alpha x\left(t\right)+(\beta_{p1}+\beta_{p2}N_{i}\left(t\right))E_{p}\left(t\right)+\beta_{s}E_{s}\left(t\right)+\beta_{c}E_{p}E_{s}-\frac{1}{2}\beta_{s}^{\prime }E_{s}^{2}\left(t\right)-\frac{1}{2}\beta_{p}^{\prime }E_{p}^{2}\left(t\right)+C\right)dt$$

where J is the total socio-economic benefit over the duration of the epidemic; αx is the direct benefit caused by the improvement in economic activity, where α is the effectiveness coefficient; and $(\beta_{p1}+\beta_{p2}N_{i}\left(t\right))E_{p}$ is the direct benefit from the epidemic prevention and control measures, including the social benefit from protecting human life and health, and the improvement of the government administration and its image as related to public health. $\beta_{s}E_{s}$ is another benefit caused by the economic stimulus in terms of production and consumption, as well as the improvement of the government’s image as related to economic development. $\beta_{s}$ is the effectiveness coefficient of economic stimulus, and $\beta_{c}E_{p}E_{s}$ denotes the benefit caused by the coupled effect of the anti-epidemic and economic stimulus, such as the welfare of public health development. An example of this development would be hospital construction. A quadratic function relation is employed to describe the relationship investment and cost [40]. Then, $\frac{1}{2}\beta_{s}^{\prime }E_{s}^{2}$ is the cost of the economic stimulus, and $\frac{1}{2}\beta_{p}^{\prime }E_{p}^{2}$ is the cost of the pandemic prevention and control measures, such as the capital and human resources used in the isolation stages, hospital posthouse construction, acid nucleic tests and vaccination, where $\beta_{s}^{\prime }$ and $\beta_{p}^{\prime }$ are the corresponding cost coefficients of both types of governmental decision-making behaviors. The constant term C is the natural socio-economic benefit without government intervention, and T is the duration of the government behavior.

In addition, as government investment amounts are limited, the constraint conditions of the optimal control problem should be satisfied as follows:

(7) $$\{\begin{array}{l} E_{p}^{2}+E_{s}^{2}\leq E^{2}\\ E_{p}\geq 0\\ E_{s}\geq 0 \end{array}$$

where the constant E denotes the limit of the upper boundary of the strengths of both pandemic prevention and control and economic stimulus.

## 5. Model Solution of Optimal Control Problem

To solve the optimal control problem, the global optimal algorithm can be utilized. However, this method requires accurate information regarding the epidemic’s duration, namely, the knowledge of the time variable T in the objective function (3). Due to the complexity of COVID-19, the end time of the epidemic cannot be predicted accurately. Thus, two strategies can be adopted to solve the optimal control problem. By assuming the constant strengths of the pandemic prevention and control and economic stimulus measures throughout the whole epidemic, the first strategy (which does not require knowledge of the epidemic’s duration) is called the static strategy, and this can be easily solved. The other strategy is called the dynamic strategy, where the duration is pre-estimated, and the investments in pandemic prevention and economic promotion are adjusted from a global optimization viewpoint. Moreover, to compare the performance of different global optimization methods, such as accuracy and usability, two algorithms are implemented, including the dynamic programming algorithm and Pontryagin’s minimum principle algorithm.

### 5.1. Solution of Static Strategy

**Figure 1:**
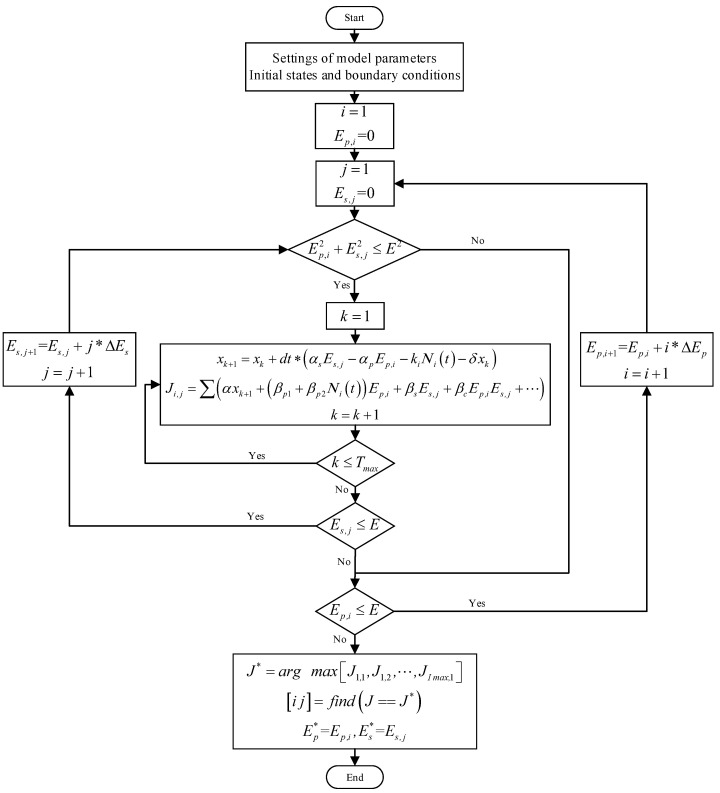
Algorithmic flowchart of the static strategy.

For the static strategy, the government carries out consistently strong epidemic prevention and control measures, as well as economic stimulus measures, throughout the entire epidemic. That is, the control variables $E_{p}$ and $E_{s}$ are both invariable. Then, the numerical solution of the optimal control problem can mainly be divided into two steps. The first step is to solve Equation (2), and the second step is to seek the optimal control variables within the allowable range by maximizing the objective function. The algorithmic flowchart is illustrated in Figure 1.

### 5.2. Solution of the Dynamic Strategy

The dynamic strategy is solved by two global algorithms, namely the dynamic programming algorithm and Pontryagin’s minimum principle algorithm.

#### 5.2.1. Dynamic Programming

Unlike the static strategy with constant government investments in epidemic control and prevention measures and economic stimulus measures, the dynamic strategy aims for a global optimal solution over the whole duration of the epidemic. This strategy is able to generate the optimal trajectory of the control variables. The discrete form of the dynamic programming strategy can be described as:

(8) $$\{\begin{array}{cc} \begin{array}{l} J_{nmax}=J_{n}\\ J_{n}=max\left\{g_{n}+J_{n+1}\right\} \end{array} & \begin{array}{l} n=T\\ n=T-1,T-2,\cdots ,1 \end{array} \end{array}$$

where $g_{n}=\alpha x_{n}+\left(\beta_{p1}+\beta_{p2}N_{i,n}\right)E_{s,n}+\beta_{s}E_{s,n}+\beta_{c}E_{p,n}E_{s,n}-\frac{1}{2}\beta_{s}^{\prime }E_{s,n}^{2}-\frac{1}{2}\beta_{p}^{\prime }E_{s,n}^{2}$ denotes the instantaneous government benefit, n denotes the time step and T is the maximum step of the epidemic’s duration.

#### 5.2.2. Effects on Optimal Strategies

According to Pontryagin’s minimum principle, the Hamilton function is defined as:

(9) $$\begin{array}{c} H\left(t,x\left(t\right),E_{p}\left(t\right),E_{s}\left(t\right),\lambda \left(t\right)\right)=\alpha x\left(t\right)+\left(\beta_{p1}+\beta_{p2}N_{i}\left(t\right)\right)E_{p}\left(t\right)+\beta_{s}E_{s}\left(t\right)+\beta_{c}E_{p}\left(t\right)E_{s}\left(t\right)\\ -\frac{1}{2}\beta_{p}^{'}E_{p}^{2}\left(t\right)-\frac{1}{2}\beta_{s}^{'}E_{s}^{2}\left(t\right)+C+\lambda \left[\alpha_{s}E_{s}\left(t\right)-\alpha_{p}E_{p}\left(t\right)-k_{i}N_{i}\left(t\right)-\delta x\left(t\right)\right] \end{array}$$

where λ is the co-state variable.

Then, the necessary conditions of the optimal solution are given as:

(10) $$\frac{\partial H}{\partial E_{p}}=\beta_{p1}+\beta_{p2}N_{i}\left(t\right)+\beta_{c}E_{s-}\beta_{p}^{'}E_{p}-\lambda \alpha_{p}=0$$

(11) $$\frac{\partial H}{\partial E_{s}}=\beta_{s}+\beta_{c}E_{p}-\beta_{s}^{'}E_{s}-\lambda \alpha_{s}=0$$

The necessary condition of the co-state has the form of:

(12) $$\dot{\lambda }=-\frac{\partial H}{\partial x}=\lambda \delta -\alpha$$

The transversality condition can be given as:

(13) λ(T)=0

By solving the differential Equation (8) with the transversality condition, we obtain:

(14) $$\lambda \left(t\right)=\frac{\alpha }{\delta }-\frac{\alpha }{\delta e^{\delta T}}e^{\delta t}=\frac{\alpha \left(e^{\delta T}-e^{\delta t}\right)}{\delta e^{\delta T}}$$

By considering Equations (7) and (8) simultaneously, we obtain:

(15) $$E_{p}^{*}=\frac{\beta_{s}^{'}(\beta_{p1}+\beta_{p2}N_{i}\left(t\right))+\beta_{c}\beta_{s}+\lambda \left(\beta_{c}\alpha_{s}-\beta_{s}^{'}\alpha_{p}\right)}{\beta_{p}^{'}\beta_{s}^{'}-\beta_{c}^{2}}$$

(16) $$E_{s}^{*}=\frac{\beta_{p}^{'}\beta_{s}+\beta_{c}\left(\beta_{p1}+\beta_{p2}N_{i}\left(t\right)\right)+\lambda \left(\beta_{p}^{'}\alpha_{s}-\beta_{c}\alpha_{p}\right)}{\beta_{p}^{'}\beta_{s}^{'}-\beta_{c}^{2}}$$

Moreover, by substituting Equation (9) into Equation (10), and considering the constraint condition, the optimal solution of both control variables $E_{p}^{**}$ and $E_{s}^{**}$ can be given as follows:

(17) $$E_{p}^{**}=min\left(\max\left(\frac{\delta e^{\delta T}\left(\beta_{s}^{'}(\beta_{p1}+\beta_{p2}N_{i}\left(t\right))+\beta_{c}\beta_{s}\right)+\alpha \left(\beta_{c}\alpha_{s}-\beta_{s}^{'}\alpha_{p}\right)\left(e^{\delta T}-e^{\delta t}\right)}{\delta e^{\delta T}\left(\beta_{p}^{'}\beta_{s}^{'}-\beta_{c}^{2}\right)},0\right),\sqrt{E^{2}-{\left(E_{s}^{*}\right)}^{2}}\right)$$

(18) $$E_{s}^{**}=min\left(\max\left(\frac{\delta e^{\delta T}\left(\beta_{p}^{'}\beta_{s}+\beta_{c}\left(\beta_{p1}+\beta_{p2}N_{i}\left(t\right)\right)\right)+\alpha \left(\beta_{p}^{'}\alpha_{s}-\beta_{c}\alpha_{p}\right)\left(e^{\delta T}-e^{\delta t}\right)}{\delta e^{\delta T}\left(\beta_{p}^{'}\beta_{s}^{'}-\beta_{c}^{2}\right)},0\right),\sqrt{E^{2}-{\left(E_{p}^{*}\right)}^{2}}\right)$$

## 6. Numerical Simulation and Results Analysis

In this section, the SIR model is first validated; then, the numerical results of the static and dynamic strategies are discussed.

The statistical data of the COVID-19 epidemic in Wuhan were employed to validate the SIR model. The data include the number of total confirmed cases, recoveries and deaths over a period of 69 consecutive days from 23 January 2020 to 31 March 2020 [41,42,43]. As the average recovery time is about 14 days, the parameter γ is set to 1/14 = 0.071. The parameter β is estimated to be 2.608 × 10^−6^ [44]. Assuming the scenario of the COVID-19 epidemic wave, the numerical simulations of the proposed optimal control problem are conducted in MATLAB for both the static and dynamic strategies. The settings of the model parameters and initial states are given in Table 1. Note that all the variables, including the state and control variables, are non-dimensional, and their values have relative sense compared to their ranges.

### 6.1. Validation of Epidemic Dynamic Model

**Figure 2:**
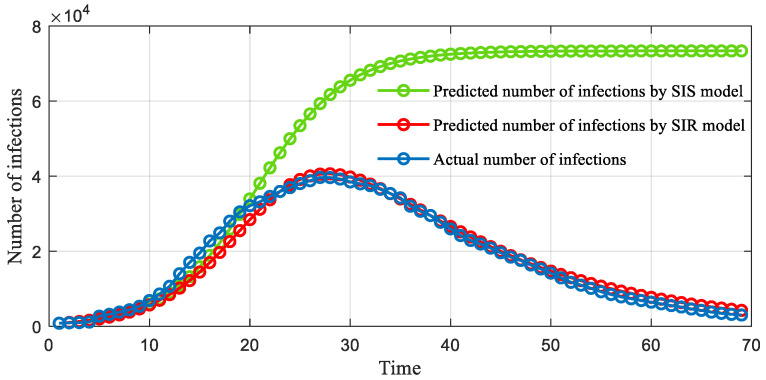
Comparison of actual and predicted infections: the green dots denote the infections predicted by the SIS model, the red dots denote the infections predicted by the SIR model and the blue dots denote the actual infections.

Figure 2 compares the predicted number of infections in each day (excluding the number of deaths and cures), as made by the SIS and SIR models, to the actual infections during the COVID-19 epidemic in Wuhan. As can be seen, the numbers in the SIR model and the actual numbers of cases are close most days, despite the slight deviations in the 10–20 day and 55–69 day range. A comparison of the SIS and SIR models indicates that in the initial stage, the two models can both achieve satisfactory predictions in this wave of the COVID-19 epidemic. However, in the middle and latter stages, the number predicted by the SIS model consistently grows until reaching the static level and significantly deviates from the actual number. The quantitative results demonstrate that the average forecasting error rate in percentage terms is no more than 13%, indicating the effectiveness of the SIR model in forecasting the number of infections of the COVID-19 epidemic.

### 6.2. Results of Static Strategy

According to the algorithmic flowchart, the numerical solution of the static strategy is generated; the results are illustrated in the following subsections.

#### 6.2.1. Optimal Solution of Static Strategy

**Figure 3:**
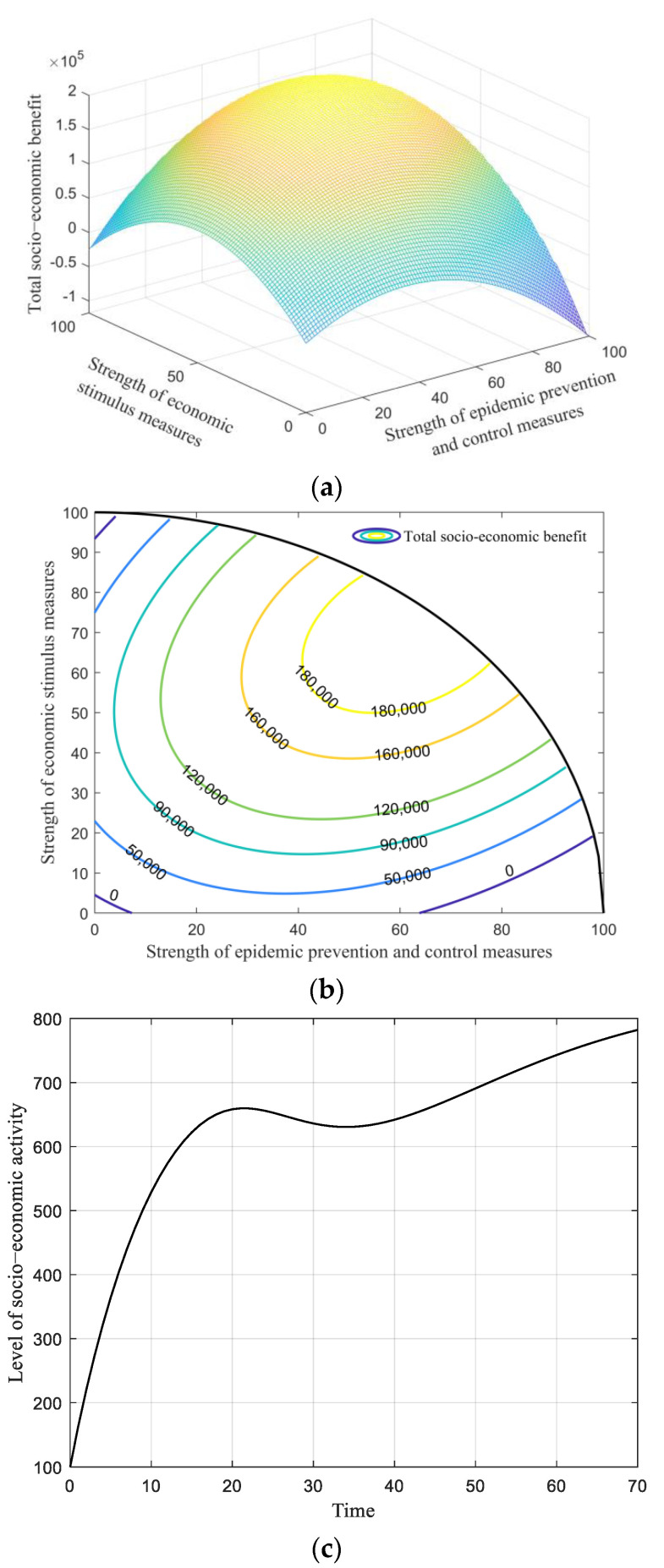

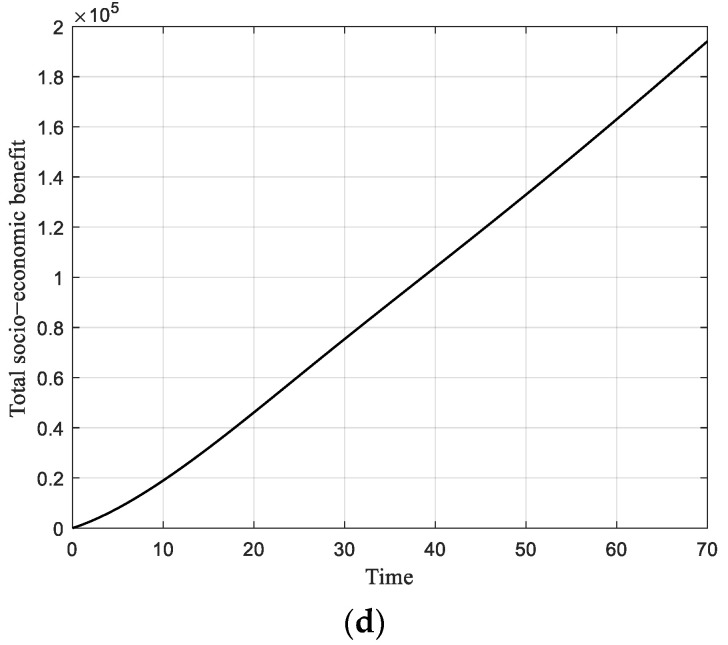
Results of static strategy. (**a**) Total socio-economic benefit with varying strengths of pandemic prevention and control measures and economic stimulus measures. (**b**) Total socio-economic benefit counters with varying strengths of pandemic prevention and control measures and economic stimulus measures. (**c**) Level of socio-economic activity, along with the time when $E_{p}$ = 62 and *E_s_* = 70. (**d**) Total socio-economic benefit, along with when $E_{p}$ = 62 and $E_{s}$ = 70.

Figure 3a shows the total socio-economic benefit with varying strengths of epidemic prevention and control measures and economic stimulus measures. The figure is shaped like a bowl with a mouth down, indicating a peak total benefit in this case. The quantitative result reveals that when $E_{p}$ and $E_{s}$ are 62 and 70, respectively, over the range of 100, the governmental total benefit reaches the maximum of 1.94 × 10^5^. This finding demonstrates that the optimal strengths of both governmental decision behaviors are around an upper-middle level, in contrast to their allowable range of 0 to 100.

Figure 3b depicts the counters of total benefit with respect to two control variables. One can observe that the investment choices between pandemic prevention and control measures and economic stimulus measures are often conflicting; increasing one would often impede the other. Figure 3c shows the level of socio-economic activity along with the time under the optimal investment strengths ($E_{p}$ = 62 and $E_{s}$ = 70); the indicator gradually climbs towards a stable level in the initial stage, but declines when the infection reaches the peak. After that, the curve grows again, with the slope reducing as time goes on, due to the accumulative effect of the consecutive infections. The total benefit, along with the time, is illustrated in Figure 3d, and the benefit almost linearly increases over the whole duration.

#### 6.2.2. Low Strength of Epidemic Prevention and Control

**Figure 4:**
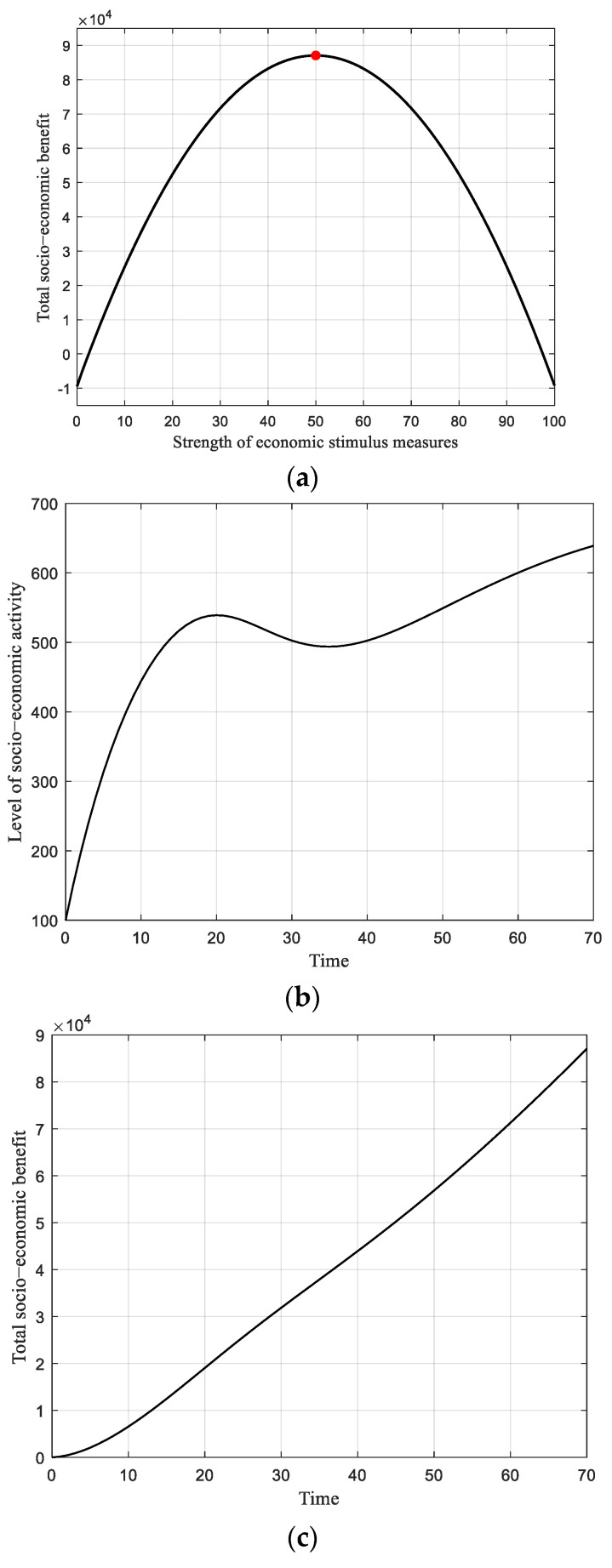
Results of static strategy with low strength of epidemic prevention and control measures. (**a**) Total socio-economic benefit versus strength of economic stimulus measures, with a low strength of pandemic prevention and control measures ($E_{p}$ = 3). (**b**) Level of socio-economic activity, along with time when $E_{p}$ = 3 and $E_{s}$ = 50. (**c**) Total socio-economic benefit, along with time when $E_{p}$ = 3 and $E_{s}$ = 50.

In the scenario in which the government chooses low-strength epidemic prevention and control measures (for example, if $E_{p}$ is 3 over the range of 100), the total socio-economic benefit is shaped like a parabola as the strength of the economic stimulus increases, as shown in Figure 4a. The total benefit rises in the first half of this stage, until reaching the peak, and then declines when the $E_{s}$ further increases. Particularly, the peak value of the total benefit is 8.7 × 10^4^ as the $E_{s}$ increases to 50, where the balance between the epidemic prevention and control measures and economic stimulus measures is realized. By comparison, the maximum total benefit here ($E_{p}$ = 3 and $E_{s}$ = 50) is significantly lower than the total benefit of 1.94 × 10^5^ in the optimal case where $E_{p}$ = 62 and $E_{s}$ = 70. This is because the lower epidemic prevention and control investment measures can lead to mass infections, which would greatly harm public health and damage the government’s image and its efforts to raise the cost in the objective function.

Figure 4a also indicates that when $E_{s}$ exceeds 50, the total benefit reduces as the economic stimulation measures are strengthened. This is due to the same reason that a looser pandemic prevention measure together with a tightened economic stimulus measure would raise the cost of public health and weaken the government’s achievements. The corresponding level of socio-economic activity is depicted in Figure 4b, where the activity gradually first rises towards a local summit and then declines to a local bottom. This occurs because the increasing infections curb socio-economic activity. When the infections gradually subside, this socio-economic level once again increases. Moreover, because the low strength of anti-epidemic measures has little impact on consumption and social mobility, the level of socio-economic activity is comparable to the optimal case (see Figure 3c), whereas the total socio-economic benefit is significantly lower than the former, as shown in Figure 4c.

#### 6.2.3. Low Strength of Economic Stimulus Measures

**Figure 5:**
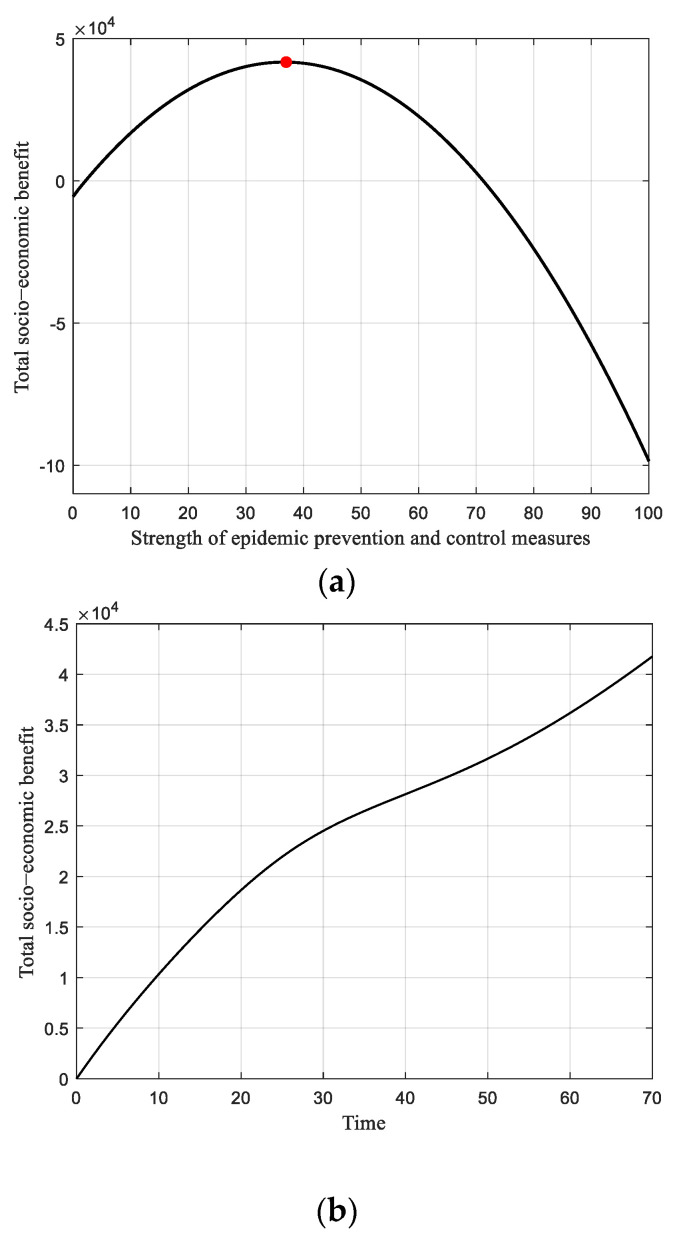

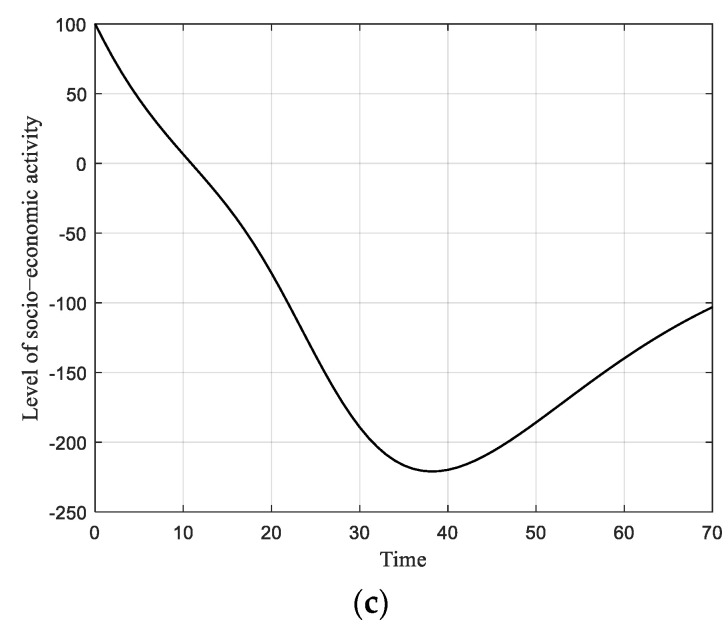
Results of static strategy with low strength of economic stimulus. (**a**) Total socio-economic benefit versus strength of pandemic prevention and control measures with a low investment in economic stimulus measures ($E_{s}$ = 3). (**b**) Trajectory of the total socio-economic benefit, along with time ($E_{p}$ = 37 and $E_{s}$ = 3). (**c**) Level of socio-economic activity, along with time ($E_{p}$ = 37 and $E_{s}$ = 3).

The scenario with a low strength of economic stimulus measures is also discussed. Figure 5a illustrates the total socio-economic benefit versus the strength of pandemic prevention and control measures with a constant low strength of economic stimulus measures ($E_{s}$ = 3 over the range of 100). Here, the peak value of the total benefit reaches 4.2 × 10^4^ when the $E_{p}$ is 37. Furthermore, one can observe that when the $E_{p}$ is higher than 71 over the range of 100, indicating significant strength in the anti-epidemic measures, the cost would exceed the marginal benefit. The total benefit then drops to a negative value, resulting in severe damage to the socio-economic activity. Compared to the results with looser pandemic prevention and control measures ($E_{p}$ = 3 and $E_{s}$ = 50), the total benefit in this scenario presents an approximate piecewise linear increasing tendency, as illustrated in Figure 5b. However, the level of socio-economic activity first experiences a decline and then growth. In the first half stage, the strict pandemic prevention measures impede the people’s mobility and economic viability, which will bring about descending socio-economic activity, while this indicator in the second half stage gradually grows, because the epidemic continuously wanes, as depicted in Figure 5c.

### 6.3. Results of Dynamic Strategy

**Figure 6:**
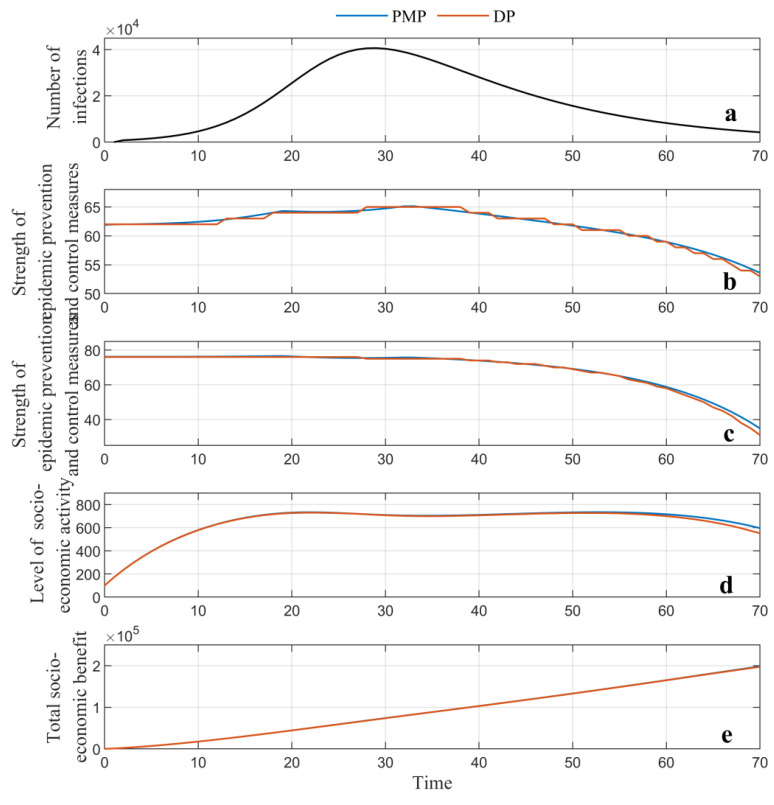
Results of the dynamic strategy generated by dynamic programming and Pontryagin’s minimum principle: (**a**) forecasted number of infections; (**b**) optimal trajectory of strength of the pandemic prevention and control measures; (**c**) optimal trajectory of economic stimulus; (**d**) optimal trajectory of the level of socio-economic activity; (**e**) total socio-economic benefit.

The dynamic strategy is simultaneously solved by two global optimization methods, namely the dynamic programming (DP) method and Pontryagin’s minimum principle (PMP) method. Different from the static strategy, where an optimal point corresponding to the maximum of the total benefit can be found, in the dynamic strategy, the optimal trajectories of the state and control variables, as well as the total benefit over the epidemic’s duration, can be generated, as depicted in Figure 6. The results show that the maximum total benefit is capable of reaching up to 1.97 × 10^5^ and 1.98 × 10^5^ for the DP and PMP methods, respectively, both of which are higher than the optimal solution (1.94 × 10^5^) of the static strategy.

To better observe and compare the changes in the critical indicators, Figure 6a shows the predicted infections over time. Figure 6b indicates that from the beginning of the epidemic to day 34, the strength of the epidemic prevention and control measures should grow slightly until reaching the highest point, in order to fight the increasing number of infective cases. One can also observe that the peak of the curves of the epidemic prevention and control is slightly delayed compared to the number of infections, mainly because the consistent containment measures can reverse the epidemic situation. The economic stimulus almost holds at the same level in this stage, as depicted in Figure 6c. Compared to its range of 100, such a level indicates a high strength of economic stimulus, which can compromise the socio-economic loss caused by the increasingly severe epidemic, so as to improve the total socio-economic benefit over the epidemic’s full duration. At this stage, the level of socio-economic activity first experiences rapid growth and then maintains a relatively static level, even showing a slight decline, due to the increasing number of infections. From day 35, the infections gradually reduce and the epidemic wanes until the end. The efforts in terms of both the pandemic prevention and control measures and the economic stimulus measures can be appropriately lowered. This will reduce the policy measures’ cost to maximize the total benefit for the whole duration of the pandemic. The two control variables obviously decline, contributing to a slight drop in the level of socio-economic activity. However, the total benefit grows sustainably, simply because the dynamic strategy enables a balance to be achieved in terms of the investment choices between the conflicting decision behaviors. This provides for the optimal total benefit for the whole duration of the pandemic.

Moreover, different results can be observed between the PMP and DP method, mainly due to the different mechanisms used to solve the optimization problem. In this study, the PMP can obtain the analytical solution of this optimal control problem; the DP could only generate the numerical solution where errors emerge in the settings of the grid partition. Additionally, a comparison between the two strategies indicates that the global optimization algorithm adjusts the strengths of governmental decision-making behaviors over the whole duration of the pandemic. This allows the government to maximize the total benefit for the whole duration of the pandemic, while the static strategy ensures that the policy measures have fixed strengths.

## 7. Sensitivity Analysis of Model Parameters

**Figure 7:**
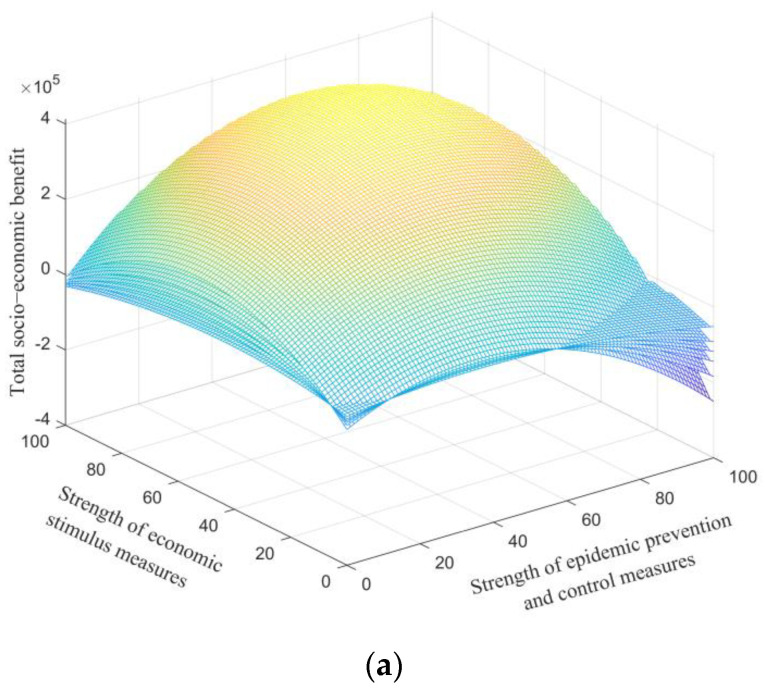

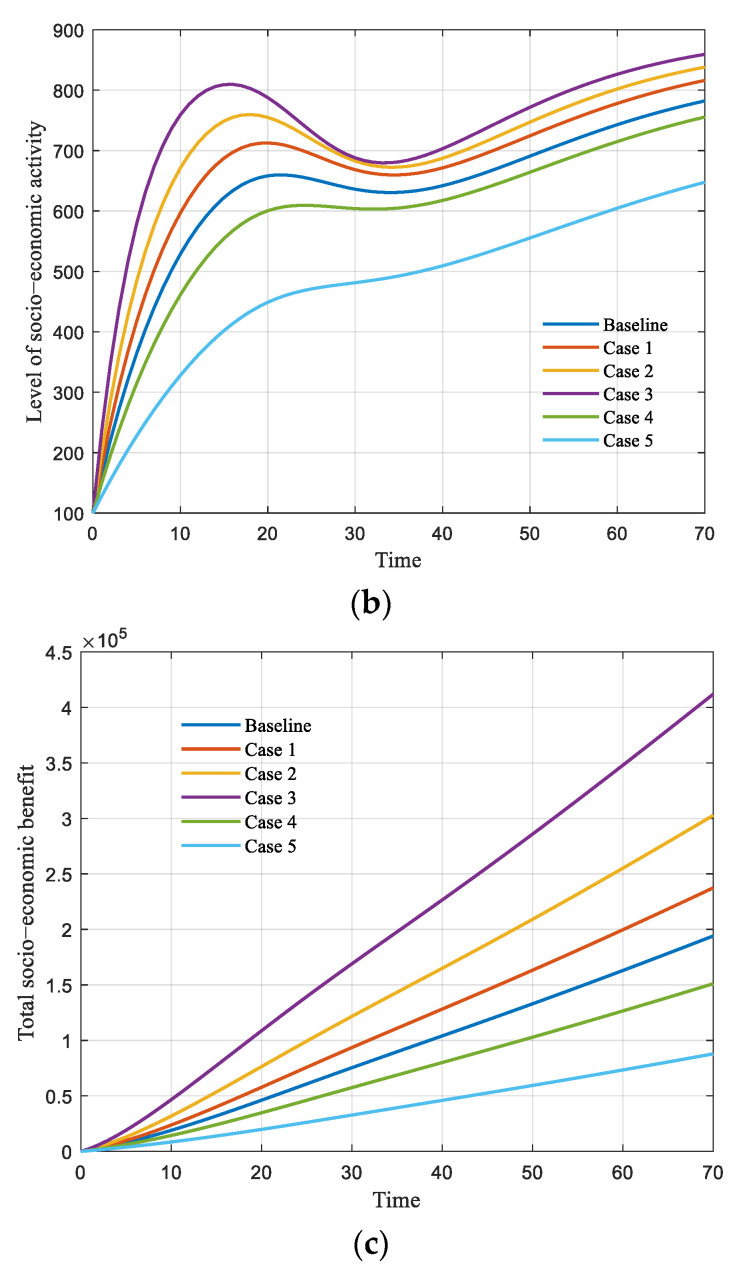
Results of static strategy in five cases with different model parameters. (**a**) Total socio-economic benefit with varying control variables in five cases. (**b**) Level of socio-economic activity along with time in five cases. (**c**) Total socio-economic benefit along with time in five cases.

**Figure 8:**
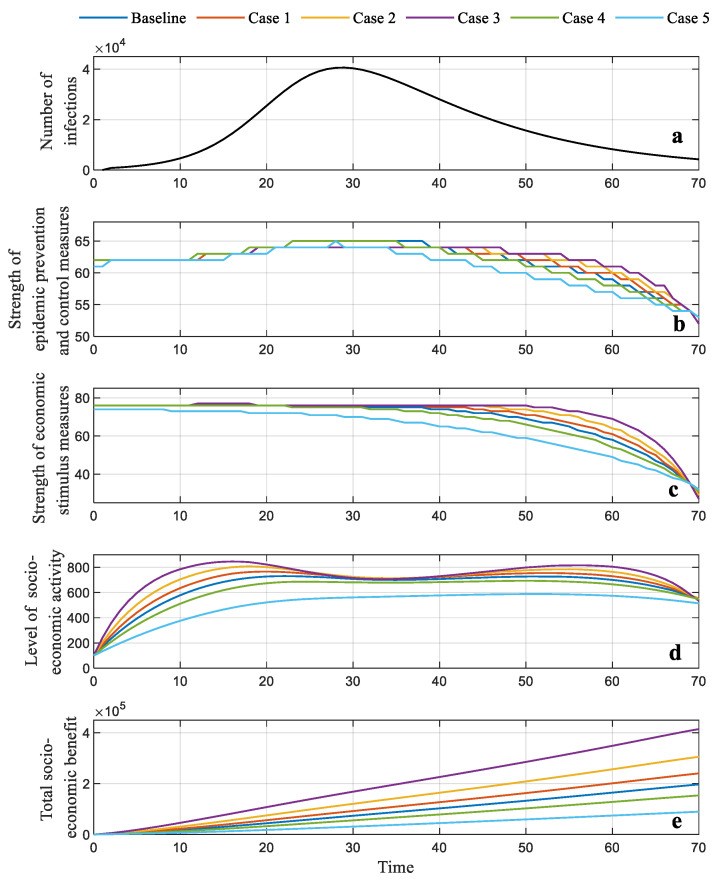
(**a**) Forecasted number of infections; (**b**) optimal trajectory of the strength of the pandemic prevention and control generated by DP; (**c**) optimal trajectory of the strength of the economic stimulation generated by DP; (**d**) optimal level of the socio-economic activity generated by DP; (**e**) total socio-economic benefit generated by DP.

**Figure 9:**
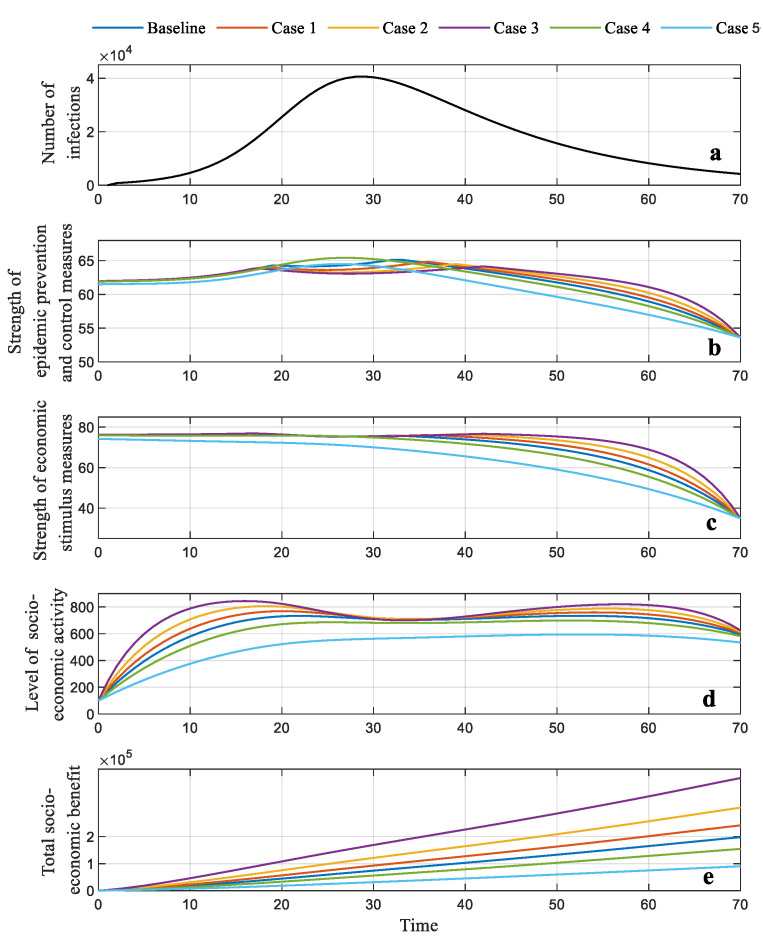
Results generated by the dynamic strategy in five cases. (**a**) Forecasted number of infections; (**b**) optimal trajectory of the strength of the pandemic prevention and control generated by PMP; (**c**) optimal trajectory of the strength of the economic stimulation generated by PMP; (**d**) optimal level of socio-economic activity generated by PMP; (**e**) total socio-economic benefit generated by PMP.

The sensitivity of all model parameters in the objective function (α, $\beta_{p1}$, $\beta_{p2}$, $\beta_{c}$, $\beta_{s}^{\prime }$, $\beta_{p}^{\prime }$) is explored. A total of five cases with the parameters expanded by 20%, 50% and 100% and reduced by 20% and 50% are discussed. All are compared to the baseline cases of the static and dynamic strategies, as presented in Section 6. The quantitative results are listed in Table 2 and illustrated in Figure 7, Figure 8 and Figure 9.

For the static strategy, optimal points corresponding to the optimal strength of the pandemic prevention and control measures and economic stimulus measures (which can be captured from Figure 7a in the five cases) fluctuate around the optimal solution of the baseline (62 and 70, respectively). These are also summarized in Table 2. The experience of the level of socio-economic activity can be divided into three sections. In the first stage (T = 0–16), the level of activity improves rapidly as the parameters expand. The indicator declines more significantly in line with the increased parameters in the second stage (T = 16–35) and then tends to maintain stable growth with a reduced slope in the final stage (T = 36–69). In Figure 7c, the total social and economic benefit approximately maintains a linear increase over the whole duration of the pandemic, and in all cases, proportionally changes in line with the variation in the parameters.

In Figure 8 and Figure 9, the forecasted number of infections is also depicted to provide a better understanding of the changes in these indicators. For the dynamic strategy, when the model parameters are raised, the trajectories of the strength of epidemic prevention and control measures in the first section (T = 0–35) experience slight growth, but the top value seems to rise gradually, as illustrated in Figure 8b and Figure 9b. The strength of the economic stimulus in the first stage changes slightly; in the second section (T = 36–69), this indicator first reduces and then increases as the parameters reduce, as illustrated in Figure 8c and Figure 9c. The level of the socio-economic activity depicted in Figure 8d and Figure 9d is improved in line with the increasing parameters. When the model parameters are reduced, the peak value of the strength of pandemic prevention and control emerges early in the first section, and declines rapidly in the following section. The total governmental benefit presents a consistent increment or reduction tendency, in line with the variation in model parameters (see Figure 8e and Figure 9e, respectively). The same conclusions can be found in Table 2.

In addition, the sensitivity of the individual parameter of the objective function is also evaluated. When each parameter is changed by 30%, the result is calculated. All results are compared to the baseline cases of the static and dynamic strategies, as presented in Section 6. The results, which are summarized in Table 3, indicate the different sensitivity. Specifically, the parameters $\beta_{p1}$ and $\beta_{s}^{'}$ present the highest impact on the socio-economic benefit in increasing and reducing the individual parameter, respectively.

## 8. Conclusions

The outbreak of a pandemic such as COVID-19 brings immense challenges to public health, as well as social and economic development. It is critical that governments all over the world realize the optimum trade-off between epidemic prevention and control measures and economic stimulus methods if they are to maximize their country’s social and economic benefits over the entire duration of the epidemic. This paper, aiming for optimal socio-economic benefit over the pandemic’s duration, uses the epidemic dynamics and optimal control theory to quantitatively discuss government investments in terms of two types of decisive behaviors. The optimal control problem is solved from both the static and dynamic strategy, and the proposed method is evaluated using the scenario of the COVID-19 epidemic. Specifically, the dynamic strategy is solved by the global algorithms of dynamic programming and Pontryagin’s minimum principle. Moreover, a sensitivity test is conducted on the parameters. The main conclusions are as follows:

(1) The SIR model can be incorporated into the optimal control problem to formulate a trade-off between the epidemic prevention and control and economic stimulus measures. Such research framework and methodology can also be applied to other epidemics.
(2) Solving the dynamic strategy requires accurate information with regard to the epidemic’s duration. The static strategy operates independent of this information, with the assumption of consistent control variables. Thus, the optimal solutions of the static and dynamic strategies are a pair of points and trajectories of control variables, respectively. Additionally, a comparison of the two strategies reveals that the optimal total social and economic benefit in the dynamic strategy is slightly greater than that of the static strategy.
(3) For the static strategy, when the government chooses a low strength of pandemic prevention and control measures (for example, 3% anti-epidemic strength), then the optimal strength of the economic stimulus is 50%. In addition, the total benefit would be significantly lowered (compared to the optimal solution) over the whole optional range. If low-strength economic stimulus measures are chosen (for example, 3% economic stimulus strength), then the optimal anti-epidemic strength is 37%. If the strength of government anti-epidemic measures exceeds the optimal value, the total social and economic benefit diminishes, and a negative value is produced. The result is severe damage to both social and economic activity and public health.

In the practice, the strength of epidemic prevention and control, to some extent, can be interpreted as the percentage of capacity of supplying anti-epidemic resources, including manpower and material resources, and the fiscal budget arranged for fighting the epidemic. As for economic interventions, the governmental stimulus measures in a policy package can be divided into different levels according to their economic effect. The strength of the economic stimulus can also be implemented as a corresponding level of policies.

(4) The solution of the dynamic strategy indicates that the government should sustain a specific strength of pandemic prevention and control measures from the beginning and then gradually improve those measures until they attain the peak value. However, an almost constant strength of the economic stimulus should initially be maintained. The peak values of both control variables are located after the summit of the infections, or the middle-to-late stage of the duration. After that, the government should properly reduce the investments in both types of decision-making behaviors.

Additionally, the comparison of both optimization algorithms demonstrated that for this optimal control problem, Pontryagin’s minimum principle algorithm is capable of acquiring the analysis solution. The dynamic programming algorithm can only generate the numerical solution.

(5) The sensitivity analysis of the overall parameters in the objective function indicates that in the static strategy, the optimal strengths of both governmental decision behaviors present a small fluctuation around the fixed point. For the dynamic strategy, the peak values of the trajectories of both control variables move with the variable parameters. This shows the time when the maximum strength is implemented, but the tendencies of the curves are almost invariable. Moreover, the influence of individual parameters on the socio-economic benefit is examined, and the results demonstrate their different sensitivities.

## 9. Limitations

This paper proposes a theoretical framework by incorporating an epidemic’s dynamics into the optimal control theory to realize the trade-off between public health and economic development. It is worth pointing out a few limitations of this research. First, the model parameters are influenced by many factors, such as the dominant regime and culture. As such, the setting of these parameters is often different from one country to another. Second, the hysteresis effect of the economic stimulus has not been considered in the modeling. Third, the application and implementation of these intervention measures in practice should be further improved. In addition, it is worth emphasizing that choosing an adequate epidemic dynamic model to predict infections becomes particularly important with the emergence of many mutant strains and the increasing number of reinfection cases. In future research, we will simulate the governmental decision-making behaviors by considering more factors.

## Figures and Tables

**Table 1:** Settings of model parameters and initial states and boundaries.

Symbol	β	γ	$k_{i}$	α	$\beta_{p}^{'}$	$\beta_{s}^{'}$	$\beta_{s}$	$\beta_{p1}$	$\beta_{p2}$	$\alpha_{s}$
Value	2.6 × 10^−8^	0.071	5 × 10^−4^	3	1.0	1.1	18	40	8.5 × 10^−5^	1.2
Symbol	$\alpha_{p}$	δ	$\beta_{c}$	*S*	*I*	*R*	$x_{0}$	T	C	E
Value	0.2	0.085	0.38	9 × 10^4^	0	0	100	70	10	100

**Table 2:** Sensitivity analysis of model parameters for static and dynamic strategies.

Case	Parameters Variation	Static Strategy		Dynamic Strategy	Comparison of Two Strategies
		Percentage Increment of Total Benefit (%)	Optimal Strengths of Decision Behaviors (*E_p_*, *E_s_*)	Percentage Increment of Total Benefit (%)	Percentage Increment (%)
Baseline	-	-	62, 70	-	-
Case 1	20% increment	22.3	63, 72	22.0	2.81
Case 2	50% increment	56.0	63, 73	55.2	0.95
Case 3	100% increment	112.2	63, 74	110.4	−10.19
Case 4	20% reduction	−22.2	62, 69	−22.0	−7.97
Case 5	50% reduction	−54.7	61, 64	−54.4	−35.02

**Table 3:** Percentage change in total socio-economic benefit when the individual parameter is changed by 30%.

Parameter	Static Strategy		Dynamic Strategy	
	30% Increment in Individual Parameter		30% Reduction in Individual Parameter	
** α **	22.47	22.44	−18.71	−19.16
** $\beta_{p1}$ **	29.31	28.29	−23.99	−23.63
** $\beta_{p2}$ **	1.03	1.04	−1.02	−1.02
** $\beta_{s}$ **	14.28	13.84	−13.17	−12.98
** $\beta_{s}^{'}$ **	−21.98	−22.02	36.03	34.53
** $\beta_{p}^{'}$ **	−15.62	−15.42	26.37	25.22
** $\beta_{c}$ **	19.98	18.88	−15.40	−15.24

## Data Availability

The datasets used in this study are available from the corresponding author on reasonable request.
